# Brain Abnormalities in Neuromyelitis Optica Spectrum Disorder

**DOI:** 10.1155/2012/735486

**Published:** 2012-12-03

**Authors:** Woojun Kim, Su-Hyun Kim, So-Young Huh, Ho Jin Kim

**Affiliations:** ^1^Department of Neurology, The Catholic University of Korea, Seoul, Republic of Korea; ^2^Department of Neurology, Research Institute and Hospital of National Cancer Center, Goyang 410-769, Republic of Korea

## Abstract

Neuromyelitis optica (NMO) is an idiopathic inflammatory syndrome of the central nervous system that is characterized by severe attacks of optic neuritis (ON) and myelitis. Until recently, NMO was considered a disease without brain involvement. However, since the discovery of NMO-IgG/antiaqaporin-4 antibody, the concept of NMO was broadened to NMO spectrum disorder (NMOSD), and brain lesions are commonly recognized. Furthermore, some patients present with brain symptoms as their first manifestation and develop recurrent brain symptoms without ON or myelitis. Brain lesions with characteristic locations and configurations can be helpful in the diagnosis of NMOSD. Due to the growing recognition of brain abnormalities in NMOSD, these have been included in the NMO and NMOSD diagnostic criteria or guidelines. Recent technical developments such as diffusion tensor imaging, MR spectroscopy, and voxel-based morphometry reveal new findings related to brain abnormalities in NMOSD that were not identified using conventional MRI. This paper focuses on the incidence and characteristics of the brain lesions found in NMOSD and the symptoms that they cause. Recent studies using advanced imaging techniques are also introduced.

## 1. Introduction

Neuromyelitis optica (NMO, also known as Devic's disease) is an idiopathic inflammatory syndrome of the central nervous system (CNS) that is characterized by severe attacks of optic neuritis (ON) and myelitis [1]. NMO had been considered as a subtype of multiple sclerosis (MS); however, after the identification of a disease-specific autoantibody, NMO-IgG, in the serum of patients with NMO [2], a dramatic conceptual change occurred. NMO-IgG binds selectively to aquaporin-4 (AQP4) [3]. AQP4 is a water-channel protein that assembles as homotetramers in cell membranes and plays a major role in fluid homeostasis of the CNS. AQP4 is found throughout the brain but is particularly abundant in the optic nerves and spinal cord [4]. In rats, AQP4 is present on astrocytic foot processes along the endothelial tight junctions at the blood-brain barrier (BBB), on the abluminal side of cerebral microvessels, within the cerebellar Purkinje cell layer and in the hypothalamus [5]. AQP4 antibody (AQP4-Ab) plays an important role in the pathogenesis of NMOSD. Probably by a mechanism involving complement-dependent astrocyte cytotoxicity, it causes leukocyte infiltration, cytokine release, and BBB disruption, leading to oligodendrocyte death, myelin loss, and neuron death [6].

The discovery of NMO-IgG or AQP4-Ab has prompted revisions of the diagnostic criteria for NMO [7] and facilitated an appreciation for the wide spectrum of this disorder (NMO spectrum disorders (NMOSDs)), which includes a proportion of patients with recurrent, isolated, longitudinally extensive myelitis (LEM) or ON as well as patients with LEM or ON associated with systemic autoimmune disease or with brain lesions typical of NMO [8] (Table 1). Furthermore, as it is increasingly recognized that some patients present with brain symptoms as their first manifestation [9–11] or develop recurrent brain symptoms without ON or myelitis, the brain abnormalities of NMOSD have been called to clinicians' attention.

 The clinical characteristics of NMOSD can overlap with other inflammatory or demyelinating CNS disorders, especially in the early stages and can cause severe disability without effective preventive treatment that differs from conventional MS treatment. Therefore, identifying the imaging characteristics of NMOSD must be an important step for the diagnosis and treatment of NMOSD. This paper focuses on the brain abnormalities of NMOSD based on their pathophysiology and clinical characteristics.

## 2. Incidence of Brain Abnormalities in NMOSD

Optic nerve and spinal cord are traditional representative regions involved in NMO. Brain MRI and orbit MRI often reveal gadolinium enhancement and edema of the optic nerve during an acute attack of ON. A spinal cord MRI obtained during the acute phases of myelitis is very likely to show a central cord lesion that extends over three or more vertebral segments. Atrophy and central cavitation are seen in later stages of the disease. The manifestation of a longitudinally extensive lesion on spinal cord MRI is one of the most characteristic neuroimaging findings for NMO. However, a lengthy cord lesion might not have fully developed in the first few days after clinical symptom onset, or it might have contracted or resolved over time [7].

 NMO has been long regarded as a disease without brain involvement, and a negative brain MRI result at disease onset was considered a major supportive criterion for the diagnosis of NMO [12]. However, a number of studies have revealed that brain abnormalities are not rare in NMO, although the inclusion and exclusion criteria are not consistent among the studies, and limitations of retrospective studies are present, including selection bias. Before the era of NMO-IgG/AQP4-Ab, brain MRI abnormalities were described in 13–46% of patients with NMO [12–21]. However, the incidence of reported brain abnormalities increased to 50–85% of patients fulfilling the 1999 NMO criteria [12] with the exception of the brain MRI criterion or revised 2006 NMO criteria (Table 2) [22–27] and to 51–79% of AQP4-Ab-seropositive patients [28, 29].

Among the brain lesions, asymptomatic lesions are more common; however, symptomatic brain involvement is also common [28]. Some NMOSD patients even present with brain symptoms as their first manifestation [9, 28, 30]. In our previous report, 15 (18%) of 83 seropositive patients with NMOSD presented with brain symptoms at disease onset [9]. Eight of them (53%) presented with encephalopathy, as evidenced by symptoms such as decreased mental status, confusion, and seizure, with or without other neurological symptoms such as dysarthria or hemiparesis. The remaining seven (47%) presented with various brainstem symptoms, including intractable hiccups, diplopia, and bulbar dysfunction without encephalopathy. Thirteen patients showed neither ON nor myelitis at the time of onset; however, all patients eventually developed one or both of these symptoms. The median time to development of ON or myelitis after the first brain symptom was 21 months (0–110 months).

A recent report described a histopathologically confirmed AQP4-Ab-positive patient who developed hemiparesis due to an isolated extensive hemispheric lesion as an initial manifestation. This paper proved that AQP4 autoimmunity indeed manifests with various brain abnormalities [10].

Pediatric patients with NMOSD also have diverse clinical presentations correlating with brain lesions. In a study analyzing 88 patients who were seropositive for NMO-IgG, MR head imaging data were available for 56 patients, and 38 (68%) of them had abnormalities that predominantly involved the periventricular areas: the medulla, supratentorial and infratentorial white matter, midbrain, cerebellum, thalamus, and hypothalamus. Twenty-six (45%) of 58 patients whose clinical information was available had episodic cerebral symptoms including encephalopathy, ophthalmoparesis, ataxia, seizures, intractable vomiting, or hiccups [31].

## 3. Characteristic Brain MRI Lesions in NMOSD

**Figure 1:**
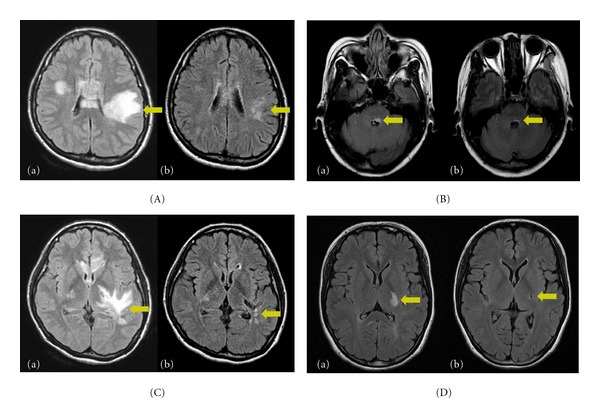
MRI findings at acute manifestation and after symptoms resolved. Fluid-attenuated inversion recovery (FLAIR) abnormalities in a patient with encephalopathy (A-a) and T2 signal abnormalities in a patient with brainstem sign (B-a) almost resolved over a few months (A-b, B-b). However, the lesions involving the corticospinal tracts, especially the posterior limb of the internal capsule (C-a, D-a) showed cavitary changes on followup MRIs (C-b, D-b) [9].

Detection of acute brain abnormalities in NMOSD is based on T2-weighted or fluid-attenuated inversion recovery (FLAIR) hyperintense signals. Interestingly, the brain lesions in T2 or FLAIR MRI often shrink or disappear on followup scans (Figure 1), and acute lesions sometimes remain as chronic T1 hypointense lesions, suggesting cell death and cavitation.

Among the brain lesions, nonspecific lesions are very commonly found. They can be dot-like or patchy, <3 cm in diameter, and located in the cerebral deep white matter, brainstem, or cerebellum [28].

However, many brain lesions in NMO or NMOSD are quite characteristic and distinct from the lesions in MS [4, 24, 28, 32, 33]. The distribution of NMO-characteristic brain lesions corresponded to sites of high AQP4 expression, adjacent to the ventricular system at any level [4]. However, other NMO-characteristic brain lesions involved where AQP4 expression is not particularly high have also been reported [28, 33].

### 3.1. Periependymal Lesions Surrounding the Third Ventricles and Cerebral Aqueduct

**Figure 2:**
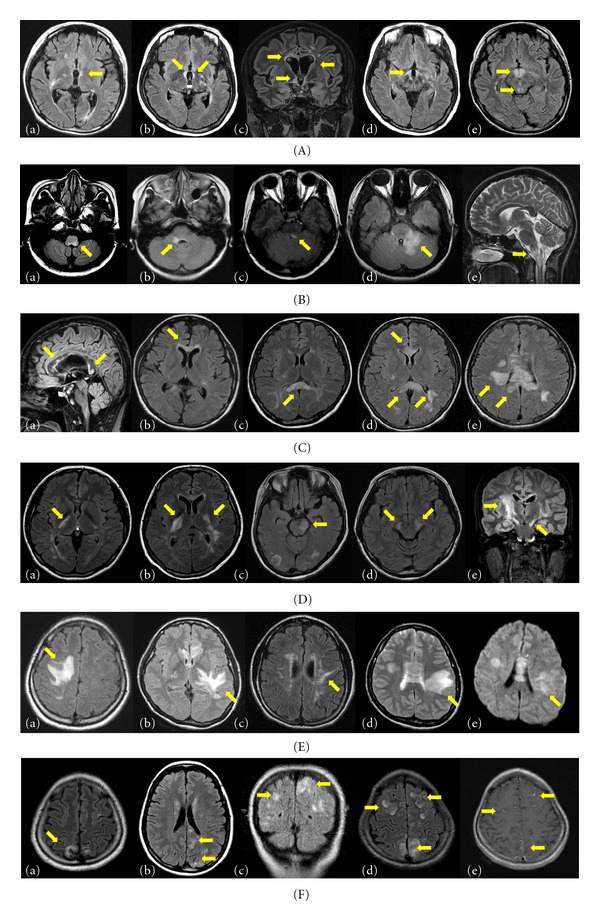
Characteristic brain abnormalities in neuromyelitis optica spectrum disorder (NMOSD) patients on T2-weighted or fluid-attenuated inversion recovery (FLAIR) MRI. (A) Periependymal lesions surrounding the third ventricle and cerebral aqueduct. These lesions can be unilateral (a) or bilateral (b) and sometime accompany with the periependymal lesions surrounding the lateral ventricles (c). They often involve the thalamus and hypothalamus (d, e). (B) Brainstem lesions adjacent to the fourth ventricle. The dorsal part of brainstem adjacent to the fourth ventricle is commonly involved (a–c). They can be edematous and form extensive lesions involving the cerebellar peduncle (d). These lesions are often contiguous with cervical lesions (e). (C) Periependymal lesions surrounding the lateral ventricles. Unlike the lesions in multiple sclerosis, NMOSD lesions are located immediately next to the lateral ventricle, following the ependymal lining in a disseminated pattern and are often edematous and heterogeneous (a). Sometimes the callosal lesions involve the entire thickness of the corpus callosum, including the splenium-like “arch bridge” (b–d). These lesions often extend into the cerebral hemisphere, forming an extensive and confluent white matter lesion (e). (D) Lesions involving the posterior limb of internal capsule and cerebral peduncle of midbrain unilaterally (a, c) or bilaterally (b, d, and e). In coronal images (e), these lesions are contiguous and longitudinally extensive following the corticospinal tracts and appear to be synonymous with a longitudinally extensive cord lesion following the descending tract. (E) Extensive and confluent hemispheric white matter lesions. They can be tumefactive (a, b) or spindle-like (c). Some lesions look like “spilled ink” along the white matter tracts. Usually they show high signal intensities on diffusion-weighted images (DWIs) (d) and an increase in apparent diffusion coefficient (ADC) values (e, paired with d), suggesting vasogenic edema. (F) Cerebral cortex involvement (a–d) and leptomeningeal enhancement (e, paired with d).

Brain MRI abnormalities in AQP4 autoimmunity are typically localized in the periependymal regions, where AQP4 is highly expressed (Figure 2(A)). Among them, the periependymal lesions surrounding the third ventricles were previously reported as “diencephalic lesions,” [4] including the thalamus and hypothalamus. The anterior border of the midbrain adjacent to the third ventricle could also be involved [4].

Hypothalamic lesions were reported in 5.0% of NMO cases in the USA [22], 5.3% in Japan [34], and 0% in Cuba [23]. Hypothalamic lesions were present in 13.3% of patients with MS as well; however, in MS, the lesions were small and triangular or lobulated in shape, whereas in NMO, they tend to be more extensive [35–37]. Some case reports have described hypothalamic lesions accompanied by various symptoms in NMO-IgG/AQP4-Ab-positive patients, including syndromes of inappropriate antidiuretic hormone secretion (SIADH) as the first manifestation [38], narcolepsy [39, 40], hypothermia, hypotension, hypersomnia, obesity [41], hypothyroidism, hyperprolactinemia, secondary amenorrhea, and galactorrhea [35].

### 3.2. Brainstem Lesions Adjacent to the Fourth Ventricle

The dorsal part of brainstem adjacent to the fourth ventricle is also commonly involved in NMOSD. These lesions are often contiguous with cervical lesions and sometimes exist solely without cervical lesions. They can be edematous and form extensive lesions involving the cerebellar peduncle (Figure 2(B)).

These lesions can be revealed by MRI without any definite clinical symptoms; however, various symptoms corresponding to the lesions may develop, such as nystagmus, dysarthria, dysphagia, ataxia, or ophthalmoplegia [30, 31, 42, 43]. Intractable hiccups, nausea, and vomiting have been reported as unique symptoms in NMO due to involvement of the cervicomedullary junction, including the perianal region, the area postrema, and the nucleus tractus solitarius [44]. Pathologic findings of area postrema lesions are tissue rarefaction, blood vessel thickening, no obvious neuronal or axonal pathology, and preservation of myelin in the subependymal medullary tegmentum [45]. Respiratory failure may occur with upper cervical cord lesions [11].

These lesions may be the first symptoms of the disease [9, 11] or herald acute exacerbation [44, 46]. Because these lesions are not detected in patients with MS, a linear medullary lesion causing intractable hiccups and nausea may distinguish NMO from MS [44]. Recently, anorexia and weight loss in patients with NMOSD with lesions involving the medulla, pons, and thalami were also reported [47].

### 3.3. Periependymal Lesions Surrounding the Lateral Ventricles

Because callosal and periventricular lesions are frequently found in MS, lesion location itself is not a unique finding that differentiates NMO or NMOSD from MS. However, the appearance of these lesions is unique. In contrast to the typical periventricular lesions in MS, which are discrete, ovoid, perpendicular to the ventricles, and perivenular extended (e.g., Dawson's fingers), NMOSD lesions are located immediately next to the lateral ventricle, following the ependymal lining in a disseminated pattern and are often edematous and heterogeneous [28, 48]. These lesions often extend into the cerebral hemisphere, forming an extensive and confluent white matter lesion (Figure 2(C)).

Lesions in the corpus callosum were described in 18.2% (4/22) of AQP4-Ab-seropositive Japanese patients with NMO, and those callosal lesions were multiple, large, and edematous with heterogeneous intensity “marbled pattern” during the acute phase [48]. Sometimes they involved the entire thickness of the corpus callosum, including the splenium-like “arch bridge.” [28, 32, 48] These lesions could be associated with some clinical symptoms, such as dysfunctions of cognition and motor coordination; however, they have not been well evaluated. Recently, diffuse and heterogeneous T2-hyperintense lesions in the splenium were reported to be characteristic of NMO [49].

### 3.4. Lesions Involving the Corticospinal Tracts

Corticospinal tract lesions have been described only occasionally in previous reports [22, 27]. However, they were the most common among characteristic brain lesions in a Korean cohort of AQP4-seropositive patients [28]. The lesions can be unilateral or bilateral and could involve the posterior limb of the internal capsule and cerebral peduncle of the midbrain. These lesions are contiguous and often longitudinally extensive, following the pyramidal tract (Figure 2(D)). They sometimes appear to be synonymous with a longitudinally extensive cord lesion following the descending tract. These lesions cause the contralateral hemiparesis, which is often very severe [9, 28]. Because these are not areas where AQP4 is highly distributed, it is unknown why these regions are frequently involved in AQP4 autoimmunity. 

### 3.5. Extensive Hemispheric Lesions

Extensive and confluent hemispheric white matter lesions are tumefactive (>3 cm in longest diameter) or configured as long spindle-like or radial-shaped signal changes following white matter tracts such as the longitudinal fasciculus. Mass effects are not usually present (Figure 2(E)). These lesions are not usually enhanced but sometimes show a “cloud-like” pattern in gadolinium enhancement [28]. It may not be easy to differentiate these lesions from other possible diagnoses, such as tumor, lymphoma, or posterior reversible encephalopathy syndrome (PRES). However, in many NMOSD cases, these lesions tend to accompany other characteristic brain lesions, which is helpful for diagnosis.

 These hemispheric lesions are likely related to vasogenic edema, and they sometimes resemble “spilled ink” following the white matter tracts. This assumption is supported by diffusion-weighted images (DWIs) and apparent diffusion coefficient (ADC) maps that show high signal intensity in both DWI and ADC maps without gadolinium enhancement [50]. Extensive hemispheric lesions in patients with NMO are reportedly related to higher antibody titers [51]. These lesions cause various symptoms such as hemiparesis, encephalopathy, and visual field defects depending on the area they involve.

### 3.6. Cortical Involvement and Leptomeningeal Enhancement

Cerebral cortex involvement and leptomeningeal enhancement in NMOSD is not common. However, the lesions are occasionally found [9, 12, 27] and are often accompanied by encephalopathy, usually in the presence of other NMO-characteristic brain lesions [9, 27] (Figure 2(F)). Recently, cortical oscillopsia without nystagmus-related cortical involvement of area V5 in a patient with AQP4-Ab-seropositive NMOSD was reported [52].

### 3.7. Enhancement Patterns

**Figure 3:**
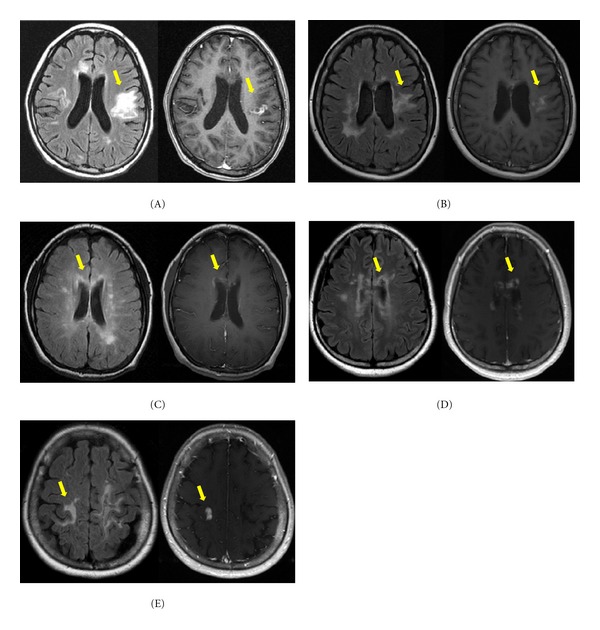
Paired fluid-attenuated inversion recovery (FLAIR) (left) and corresponding gadolinium-enhanced (right) axial images of patients who showed enhancement. Enhancement observed in extensive hemispheric lesions (A, B, and E) and periependymal lesions surrounding the lateral ventricles (C, D). The lesions reveal multiple patchy enhancement patterns with blurred margins, showing typical “cloud-like enhancement”. One exceptional lesion reveals well-marginated nodular enhancement at an extensive hemispheric lesion (E) [28].

Most brain lesions in patients with NMOSD are not enhanced, although 13–56% of patients showed at least one enhancing brain lesion on brain MRI during their disease course [9, 26, 27]. In a previous report describing characteristic brain lesions in patients with NMOSD, extensive hemispheric lesions, and periependymal lesions surrounding the lateral ventricles were most commonly enhanced, while only one corticospinal tract lesion and one nonspecific white matter lesion showed enhancement [9] (Figure 3).

The most prominent type of enhancement is “cloud-like enhancement,” which appears as multiple patches of enhancing lesions with blurred margins [26]. Well-marginated nodular enhancement is rarely found [9, 27]. The mechanism of this characteristic enhancement pattern is not known. Ito et al. suggested that the primary breakdown of the BBB due to loss of AQP4 from the astrocyte foot processes may allow for secondary influx of humoral or cellular immune components that attack the adjacent brain regions, and the resultant sustained chained inflammation could be a cause of cloud-like enhancement [26].

## 4. Brain Abnormalities in the Diagnosis of NMO and NMOSD

Due to the growing recognition of brain abnormalities in NMOSD, such abnormalities have been included in the diagnostic criteria or guidelines of NMO and NMOSD. The NMO criteria revised in 2006 include the conservative criterion “brain MRI at onset does not meet the diagnostic criteria for MS.” [7] However, an international task force recommended diagnostic criteria for NMO that included more detailed criteria for brain MRI findings [53]. The criteria for the NMO spectrum included ON or myelitis associated with brain lesions typical of NMO; these were hypothalamic, corpus callosal, periventricular, or brainstem lesions [8] (Table 3).

However, differential diagnosis of NMO or NMOSD is still a challenge. Patients who are seropositive for AQP4-Ab can manifest an isolated brain abnormality without ON or myelitis, and some patients with NMOSD show brain MRI abnormalities satisfying the Barkhof criteria for dissemination in space [22, 28, 30, 54]. Thus, applying the Barkhof criteria without consideration of the configuration of the lesions can lead to misdiagnosis. 

A study using 7-T MRI showed that in contrast to MS plaques, which were nearly exclusively centered on a small vein (92%), white matter changes in patients with NMOSD were nonspecific in appearance and were only infrequently neighbored by a blood vessel (35%). Characteristic hypointense rims were very rarely detectable in NMOSD (2%) compared with MS (23%), and cortical pathology was absent in NMOSD [55]. Those MRI features of white matter and the absence of cortical gray matter findings substantially differentiate NMOSD from MS and can be used as a potential marker to distinguish these two entities [55]. Other study using double inversion recovery sequence and a semiautomatic software (Freesurfer) revealed that the absence of NMO differs from MS, where cortical lesions and atrophy are frequently found, even in early disease phases. Thus, MRI analysis of the cortex may be a potential diagnostic tool, especially in ambiguous cases [56].

Previous studies have reported acute disseminated encephalomyelitis (ADEM) or PRES in patients with NMOSD [57, 58]. Patients with ADEM sometimes show brain lesions very similar to confluent white matter lesions in NMOSD [59]. Neuroimaging of ADEM is characterized by large demyelinating lesions in the spinal cord extending over several segments and/or in the brain, often involving astrocyte water channels [59]. Some patients experience multiple events, called “recurrent disseminated encephalomyelitis (RDEM),” “multiphasic disseminated encephalomyelitis (MDEM),” or “multiphasic ADEM.” [60, 61] The similarities to NMO raise the expectation that other specific autoantibodies will be identified to explain ADEM and its variations [59]. The presence of diffuse subcortical edema preferentially affecting the parietooccipital regions indicates PRES. Considering this finding and the recently increased recognition of brain manifestations in patients with NMOSD, it might be more appropriate to consider that such brain abnormalities are one manifestation of AQP4 autoimmunity rather than a cooccurrence of NMOSD and ADEM or PRES.

Two independent reports recently compared AQP4-Ab-seropositive and -seronegative groups [54, 62]. In one study, the McDonald criteria were less frequently fulfilled on brain MRI in seropositive patients (5.6% versus 33.3%). Juxtacortical and corpus callosal lesions were also less common in this group (16.7% versus 46.7%; 5.6% versus 46.7%). Hypothalamic and periaqueductal involvement were not seen in seronegative patients [54]. However, in the other study, the frequency of neither supratentorial nor infratentorial brain lesions differed [62].

NMO has occasionally been associated with other autoimmune diseases, including hypothyroidism, Sjögren's syndrome (SS), systemic lupus erythematosus (SLE), pernicious anemia, ulcerative colitis, primary sclerosing cholangitis, rheumatoid arthritis, mixed connective tissue disorders, and idiopathic thrombocytopenic purpura [1]. In a study analysing brain abnormalities in SS with recurrent CNS manifestations, AQP4-Ab was positive in six of eight patients tested, and their brain MRI findings were not different from characteristic brain lesions reported in NMOSD [63]. In other study analysing 22 consecutive SS patients with CNS manifestations, 31.8% of them were positive for AQP4-Ab, and the abnormalities in the cerebrum and brainstem, as well as the optic nerve and the spinal cord, were more commonly found in AQP4-Ab-positive patients than in Ab-negative patients [64].

## 5. Advanced MRI Techniques in NMOSD

Recent advances in MRI techniques using diffusion tensor image, MR spectroscopy, and voxel-based morphometry are revealing new findings related to brain abnormalities in NMOSD that have not been found on conventional MRI.

 Diffusion tensor images with tract-based spatial statistical analysis revealed that multiple white matter tracts were involved, including the pyramidal tract, optic radiation, and corpus callosum. This involvement was likely related to both demyelination and Wallerian degeneration [65]. Recently proposed framework for detecting longitudinal change in diffusion MRI using multivariate statistical testing on tensors could provide useful tools to detect changes in the normal-appearing white matter (NAWM) that are related with physical status outcome [66].

 MR spectroscopy findings were normal in both NAWM and normal-appearing grey matter (NAGM) in NMO patients for the main metabolic parameters such as N-acetyl-aspartate (NAA), choline and myoinositol, corresponding to axonal loss, inflammations and gliosis, respectively. This is clearly different from the findings in MS, where NAA is frequently decreased and choline increased, even in NAWM, suggesting infraradiological destruction, including axonal loss [67, 68].

 Voxel-based morphometry (VBM) provides means to investigate the structural changes of whole brain in an automated technique. A recent study analysing grey matter atrophy using VBM revealed that relapsing-remitting MS (RRMS) had significant grey matter loss in bilateral thalami, caudate, left parahippocampal gyrus, right hippocampus, and insula, suggesting axonal degeneration of the deep grey matter in RRMS compared to NMO [69]. The decrease of global and focal WM in NMO patients, including brainstem, corticospinal tracts, corpus callosum, and superior and inferior longitudinal fascicles, was revealed to be correlated to their cognitive impairment [70]. Another study on occult damage in the brain of patients with NMO used a multiparametric MRI approach and revealed selective alteration of the optic pathways and the lateral geniculate nuclei in track-based spatial statistics, significantly different diffusion tensor imaging values in the NAWM in the patients with NMO versus the healthy controls and a significant density and volume reduction of the sensorimotor and visual cortices in VBM analysis. These findings suggest structural damage in the brain of patients with NMO predominantly involving regions connected with the motor and visual systems [71].

## 6. Conclusion

Brain abnormalities in NMOSD are more common than previously thought, and some patients even manifest brain symptoms as their first presentation. Although many of the brain lesions in NMOSD are nonspecific, their characteristic locations and configurations are helpful in the diagnosis of NMO or NMOSD. The underlying pathomechanisms of various brain lesions in NMOSD are still unknown, and further studies are needed. An understanding of diverse brain manifestations is now crucial for early and correct diagnosis of NMOSD. 

## Figures and Tables

**Table 1:** NMO spectrum [8].

NMO	
Limited form of NMO	
Idiopathic single or recurrent events of longitudinally extensive myelitis (≥3 vertebral segment spinal cord lesions seen on MRI)	
Optic neuritis: recurrent or simultaneous bilateral	
Asian optic-spinal multiple sclerosis	
Optic neuritis or longitudinally extensive myelitis associated with systemic autoimmune disease	
Optic neuritis or myelitis associated with brain lesions typical of NMO (hypothalamic, corpus callosal, periventricular, or brainstem)	

**Table 2:** Revised criteria for the diagnosis of NMO [7].

Optic neuritis	
Acute myelitis	
At least two of the following three supportive criteria	
Contiguous spinal cord MRI lesion extending over at least three vertebral segments	
Onset brain MRI not meeting the diagnostic criteria for MS	
NMO-IgG seropositivity status	

**Table 3:** Diagnostic criteria of NMO^a^ [53].

Major criteria (all criteria are required but may be separated by an unspecified interval)	
(i) Optic neuritis in one or more eyes	
(ii) Transverse myelitis, clinically complete or incomplete, but associated with radiological evidence of spinal cord lesion extending over three or more spinal segments on T2-weighted MRI images and hypointensity on T1-weighted images when obtained during acute episode of myelitis	
(iii) No evidence for sarcoidosis, vasculitis, clinically manifest systemic lupus erythematosus or Sjögren's syndrome, or other explanation for the syndrome	

Minor criteria (at least one must be fulfilled)	

(1) Most recent brain MRI scan of the head must be normal or may show abnormalities not fulfilling Barkhof criteria used for McDonald diagnostic criteria, including^b^.	
(i) non-specific brain T2 signal abnormalities not satisfying Barkhof criteria as outlined in McDonald criteria	
(ii) lesions in the dorsal medulla, either in contiguity or not in contiguity with a spinal cord lesion	
(iii) hypothalamic and/or brainstem lesions	
(iv) “Linear” periventricular/corpus callosum signal abnormality, but not ovoid, and not extending into the parenchyma of the cerebral hemispheres in Dawson finger configuration	
(2) Positive test in serum or CSF for NMO-IgG/aquaporin-4 antibodies	

^a^These criteria exclude limited or inaugural syndromes that may be NMO, such as recurrent transverse myelitis with longitudinally extensive spinal cord lesions or recurrent ON; further study is warranted to clarify their relationship with NMO, especially in the setting of seropositivity for NMO-IgG/AQP4 antibodies.

^b^Periodic surveillance with brain MRI scanning is necessary to monitor for the emergence of new lesions that may lead to a revised diagnosis.
